# Preparation, characterization and performance analysis of sustainable kaolinitic clay-based ceramics incorporating ternary blends of steel slag, coal fly ash and waste glass bottle-derived powder

**DOI:** 10.1039/d6ra00763e

**Published:** 2026-03-17

**Authors:** Madeniyet Yelubay, Dias Tolegenov, Sabit Maussumbayev, Nurdana Kanasheva, Gulzat Aitkaliyeva, Vladimir Mokichev, Stepan Denisov, Sergey Tsvetkov, Anton Kasprzhitskii, Georgy Lazorenko

**Affiliations:** a Department of Chemistry and Chemical Technology, Toraighyrov University Pavlodar 140000 Kazakhstan Yelubay.m@tou.edu.kz diastolegenov1992@gmail.com mausumbaevsabit@gmail.com kanashevanur94@gmail.com; b Department of Chemical and Biochemical Engineering, Satbayev University Almaty 050013 Kazakhstan g.aitkaliyeva@satbayev.university; c Climate Center, Novosibirsk State University Pirogov Street, 2 Novosibirsk 630090 Russia v.mokichev@g.nsu.ru s.denisov@g.nsu.ru s.tsvetkov@g.nsu.ru a.kasprzhitskii@nsu.ru g.lazorenko@nsu.ru

## Abstract

The valorization of industrial solid wastes into construction materials represents an important pathway toward resource efficiency and carbon reduction in the building sector. In this study, sustainable kaolinitic clay-based ceramics were developed using ternary blends of steel slag (SS), coal fly ash (CFA), and recycled waste glass bottle-derived powder (WGBP). The effects of WGBP content and firing temperature on phase evolution, microstructural development, densification behavior, and key physico-mechanical properties were systematically investigated. The results show that at intermediate temperatures (1000–1100 °C), the addition of 5 wt% WGBP promotes liquid-phase sintering, leading to enhanced densification, reduced water absorption, and compressive strengths up to 44 MPa, whereas higher glass contents at elevated temperature induce over-fluxing and pore entrapment, reducing strength despite comparable density. XRD, FTIR, and SEM analyses confirm the progressive vitrification and structural reorganization of the aluminosilicate matrix. The sustainability assessment identifies the 5 wt% WGBP formulation as the most balanced option, combining adequate mechanical performance with lower energy demand and CO_2_ emissions. Overall, the proposed approach provides a technically viable and resource-efficient route for the integrated utilization of multiple industrial wastes in construction ceramics.

## Abstract 2

Production of sustainable sintered composite materials based on steel slag, coal fly ash, clay, and waste glass powder at 1000–1200 °C.

## Graphical abstract

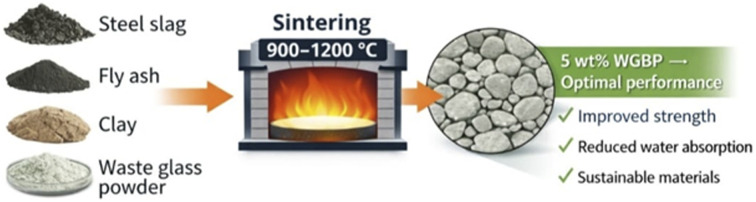

## 1. Introduction

The effective management of inorganic industrial by-products is a significant priority for resource-intensive economies, particularly in regions undergoing rapid industrial development.^1–3^ The Republic of Kazakhstan, as a major global producer of steel and a nation reliant on coal-fired power generation, exemplifies this challenge. The national metallurgical and energy sectors generate millions of tons of solid residues annually, primarily steel slag (SS) and coal fly ash (CFA).^4,5^ In Kazakhstan, ∼19 million tons of CFA (10% of industrial waste) is generated yearly.^6^ By 2024, over 300 million tons occupied ∼8500 ha, contrasting sharply with a processing rate of just 0.7%.^6,7^ With an annual domestic crude steel production in excess of 4 million tons,^8^ the associated generation of steel slag is significant, typically amounting to 10–15 wt% of the crude steel produced.^9^ Current disposal practices, predominantly involving accumulation in ash dumps and slag heaps, not only lead to significant land occupation and landscape degradation but also represent a substantial loss of potential secondary raw materials within the national economy.^10–12^ Furthermore, the environmental risks associated with the long-term storage of these wastes, including dust generation^13–15^ and potential leaching of residual heavy metals into soils,^16–18^ surface water and groundwater,^19,20^ necessitate the development of advanced, locally viable valorization pathways. Consequently, transforming these abundant, low-cost domestic waste streams into value-added, engineered materials is a critical research objective aligned with strategic goals for sustainable industrial development, waste minimization, and the principles of a circular economy.

Ceramic technology offers a promising route for the consolidation and upcycling of such inorganic solid wastes. The vitrification and sintering of waste-derived powders can immobilize potentially toxic elements and transform them into durable, monolithic materials suitable for building applications, such as tiles,^21,22^ pavers,^23,24^ or architectural ceramics.^25,26^ Research on SS- and CFA-based ceramics has demonstrated their technical feasibility, highlighting the influence of composition and sintering conditions on densification, microstructure and mechanical strength.^27–29^ However, the chemical and phase composition of these systems is often dominated by refractory phases (*e.g.*, calcium silicates and iron oxides in SS; mullite and quartz in CFA), which can require high sintering temperatures (>1100 °C) for adequate densification, thereby increasing energy costs and limiting the economic viability of the process.^30,31^

The introduction of fluxing agents is a well-established strategy to lower the sintering temperature and promote liquid-phase sintering in ceramics. Waste glass, rich in silica and fluxing oxides (Na_2_O, CaO), acts as an effective and low-cost flux, reducing viscosity and enhancing particle rearrangement and pore elimination at lower temperatures.^32–34^ Previous studies have investigated the incorporation of glass cullet into clay-based ceramics or as a partial substitute in some waste systems. The study by Kim *et al.*^35^ demonstrates that LCD process waste glass (LPWG) can effectively replace feldspar as a flux in ceramic tiles by up to 40 wt%, due to its optimal viscosity of 10^5^–10^5.6^ dPas at sintering temperatures (1100–1150 °C). This substitution enabled the production of dense bodies with near-zero water absorption and a stable mullite content of 4–6 wt%, highlighting LPWG's potential for sustainable recycling in the tile industry. Bohn *et al.*^36^ Demonstrate that waste soda-lime glass can serve as an effective flux in ceramic pavers, enabling the reduction of firing temperatures. Incorporating 40–60 wt% of waste glass lowered the required sintering temperature to 900–950 °C, producing pavers with high compressive strength (up to 61.4 MPa) and low water absorption (as low as 0.42%). Adediran *et al.*^37^ demonstrate that glass wool waste can be effectively recycled as a fluxing agent in building ceramics. Incorporating 10 wt% of glass wool significantly enhanced the densification and properties of ceramics derived from quartz-feldspar sand (QFS), copper slag (CS), and kaolin. The QFS-based ceramic with 10% glass wool sintered at 950 °C achieved a high compressive strength of 117 MPa and low water absorption of 2%, while also allowing for a substantial reduction in the required firing temperature compared to traditional fluxes. Mustafi *et al.*^38^ Demonstrate that rice husk ash (RHA, 40–50 wt%) and waste glass powder (WGP, 15–25 wt%) can be effectively utilized as primary silica sources and fluxes, respectively, in the production of glass-ceramic tiles. Sintering the glass frit within at optimal temperature of 925 °C resulted in tiles with excellent properties, including a high modulus of rupture (95.25 MPa), low water absorption (0.26%), and high glossiness (48.3%). The role of waste glass as a fluxing agent has been similarly confirmed in the works of Conte *et al.*^39^ Baccarin and Bragança,^40^ and Ruan *et al.*^41^ However, a critical research gap persists concerning the systematic integration of post-consumer waste glass bottle powder (WGBP) into SS/CFA containing ceramic matrices. The specific effects of WGBP on the phase evolution, microstructure development, and the resultant physico-mechanical and surface properties (*e.g.*, wettability) in such ternary systems are not well quantified. Furthermore, the interaction between the replacement ratio of this multicomponent blend and the sintering temperature requires detailed optimization to balance performance and energy efficiency.

Therefore, this study was designed to (i) fabricate sustainable ceramics from a dual waste system of SS and FA, incorporating WGBP as a fluxing agent; (ii) systematically investigate the individual and synergistic effects of WGBP content and sintering temperature on the phase evolution, microstructural development, and densification kinetics; (iii) comprehensively evaluate the consequent physico-mechanical properties, including compressive strength, volume shrinkage, bulk density, water absorption, and surface wettability; and (iv) elucidate the critical processing-structure–property relationships governing the performance of these waste-derived ceramic materials. The findings are expected to provide a scientific and technical framework for developing energy-efficient, high-performance ceramics, while offering an integrated solution for managing multiple solid waste streams.

## 2. Experimental procedure

### 2.1 Raw materials

**Fig. 1:**
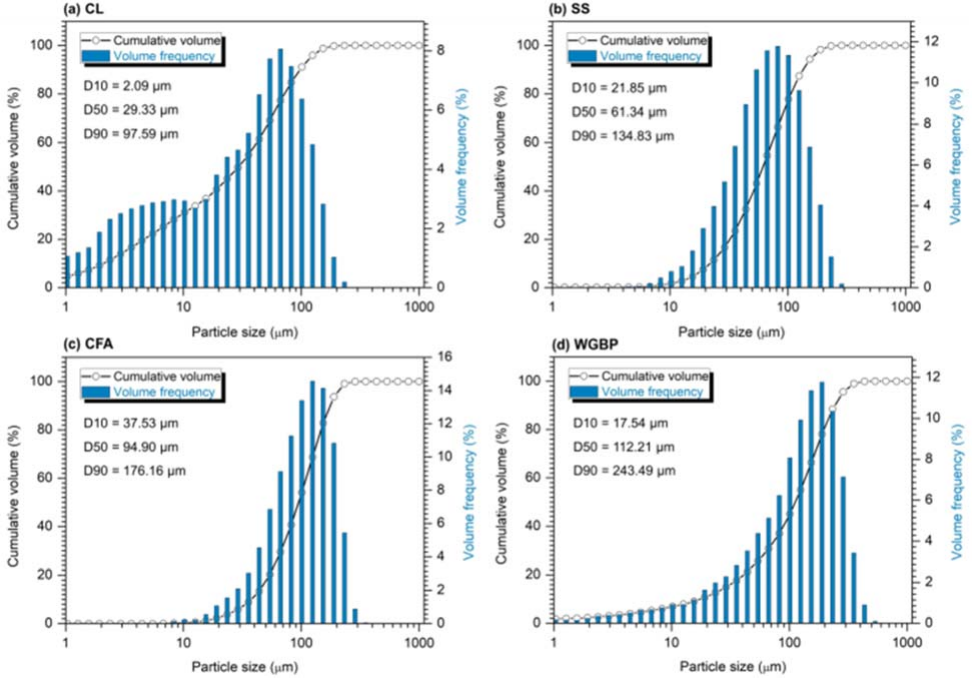
Particle size distributions of raw materials: (a) CL, (b) SS, (c) CFA, (d) WGBP.

**Fig. 2:**
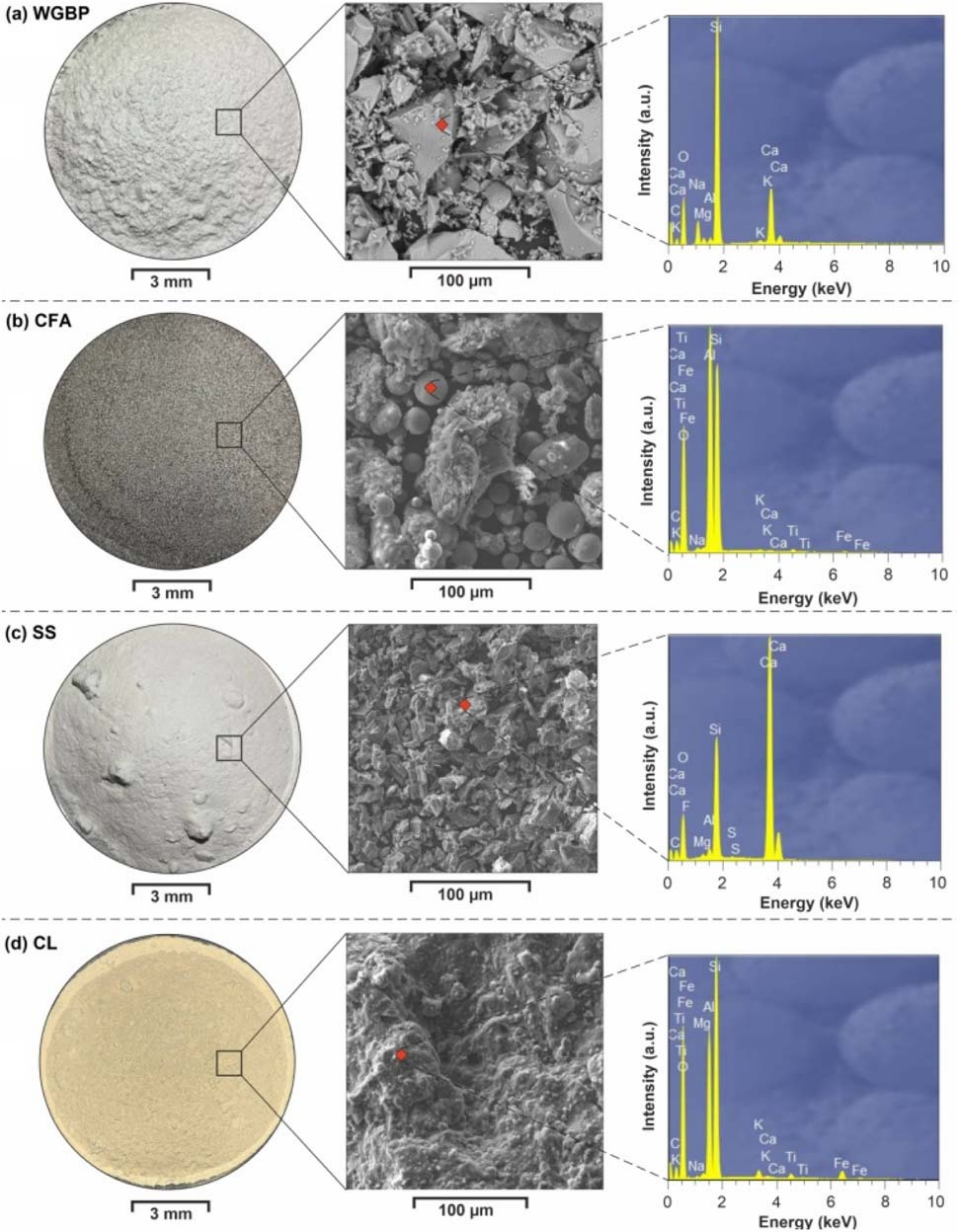
Optical macrographs, SEM images and point EDS spectrum of raw materials: (a) WGBP, (b) CFA, (c) SS and (d) CL.

The clayey raw material was refractory clay (CL) sourced from the Kemertuz deposit in Kazakhstan's Pavlodar Region. The steel slag was provided by KSP Steel (Pavlodar, Kazakhstan).^42^ The fly ash was a by-product of Ekibastuz coal combustion obtained from Pavlodar thermal power stations. The waste glass powder was derived from post-consumer soda-lime glass bottles (local manufacturers), which were washed, dried, and subsequently crushed. The raw materials were initially dried to constant mass at 100 °C (UT-4603 oven, Xieli International Trading Co., China). Subsequent milling was performed in a planetary ball mill (BM6 Pro, POWTEQ, China) using zirconium dioxide jars and balls at 400 rpm for 5 min. The resulting powder was sieved through a 0.05 mm mesh. Granulometric analysis, as shown in Fig. 1, was performed using a laser diffraction particle analyzer SALD 2101 (Shimadzu Corp., Japan). A representative sample was suspended in 50% ethanol. The suspension was sonicated (1 min, 22 kHz, 800 W) and mixed intensively (2 min). Subsequently, an aliquot was analyzed in the instrument's measurement cell according to ASTM D4464-15. The median particle sizes of CL, SS, CFA, and WGBP were measured as 29.3, 61.3, 94.9, and 112.2 µm, respectively (Table 1 and Fig. 1). A TESCAN MIRA analytical scanning electron microscope (TESCAN, Czech Republic) with an energy-dispersive X-ray microprobe was used to acquire the SEM images shown in Fig. 2, at 20 kV accelerating voltage and 1400 pA beam current. SEM-EDS analysis indicates that the raw materials exhibit distinct particle morphologies and heterogeneous microstructures, ranging from angular and platy particles to more porous and agglomerated textures, reflecting their different origins. Elemental spectra confirm that all materials are predominantly composed of aluminosilicate phases with varying amounts of Ca-, Fe-, Mg-, and alkali-bearing components, highlighting their potential reactivity and functional roles when incorporated into ceramic systems. X-ray fluorescence (XRF) analysis was performed to determine the chemical composition of the major phases, using an Applied Research Laboratories ARL-9900-XP spectrometer (Thermo Electron Corporation, USA). Sample preparation involved calcining the pre-dried powders at 1000 °C for 2.5 hours. The calcined product was then mixed with a non-wetting lithium borate flux (66.67 wt% lithium tetraborate, 32.83 wt% lithium metaborate, 0.5 wt% lithium bromide) in a 1 : 9 (sample : flux) ratio.

**Table 1:** Bulk density, median particle size and specific surface area of raw materials

Raw material	CL	SS	CFA	WGBP
Bulk density (g cm^−3^)	0.97	1.53	0.91	1.74
Median particle size (µm)	29.3	61.3	94.9	112.2

**Fig. 3:**
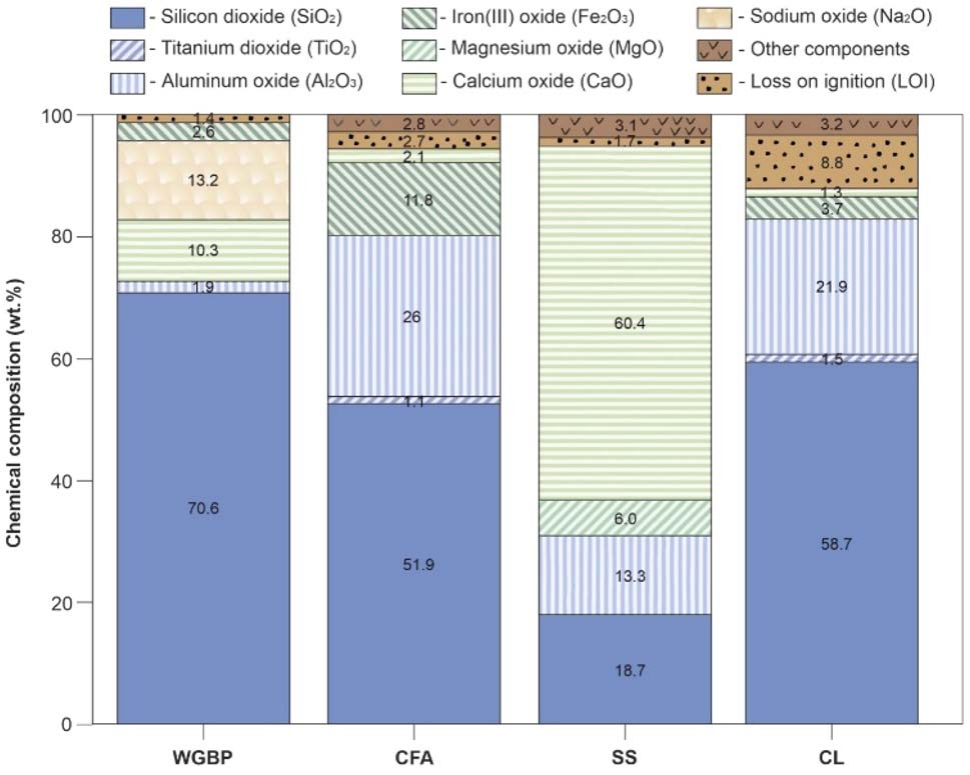
Chemical composition of raw materials.

**Fig. 4:**
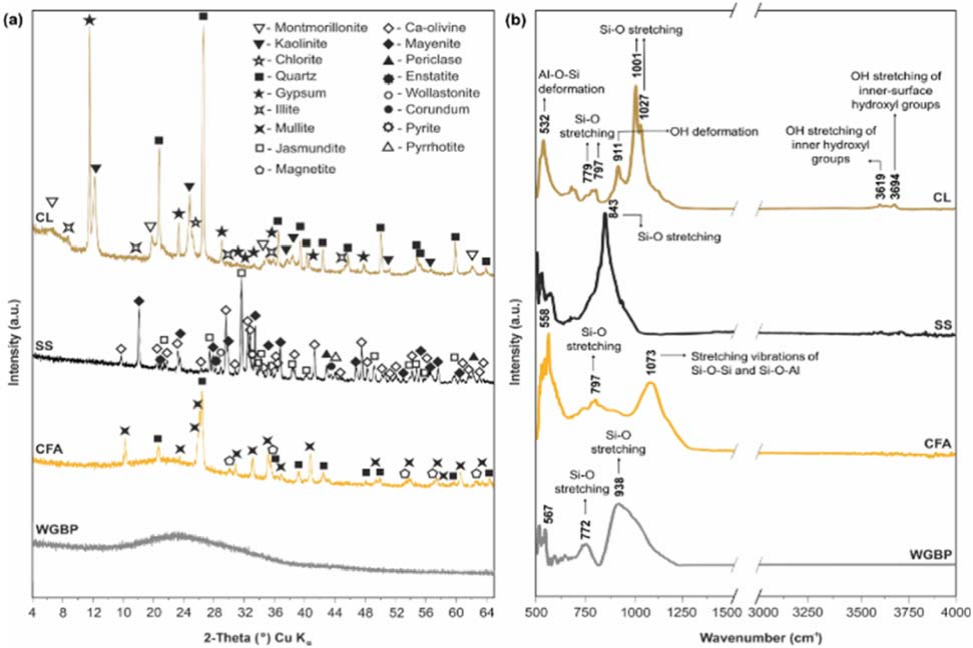
(a) Diffraction patterns by XRD and (b) FTIR spectra for raw materials.

**Fig. 5:**
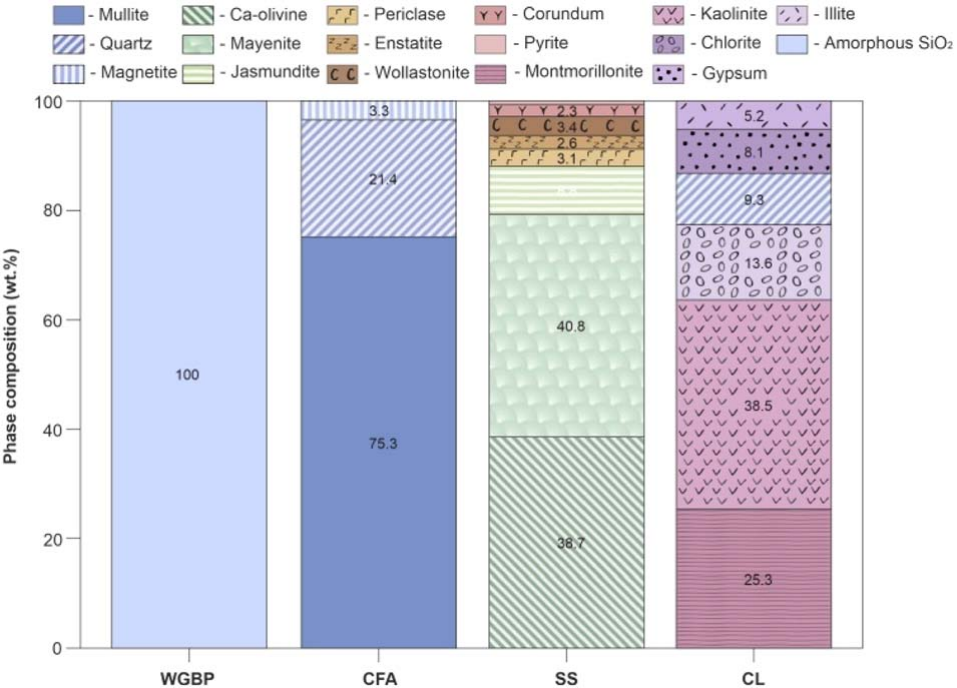
Phase composition of raw materials.

Fusion was carried out in platinum crucibles using a Lifumat-2.0-Ox automatic fusion machine (Linn High Therm, Germany) to produce homogeneous glasses for XRF analysis. XRF results show that the raw materials differ markedly in oxide composition (Fig. 3). CFA and CL are aluminosilicate materials rich in alumina (Al_2_O_3_)^43^ and silica (SiO_2_). WGBP is characterized by elevated SiO_2_ (70.6 wt%), Na_2_O (13.2 wt%), and CaO (10.3 wt%) contents, while SS is dominated by CaO (60.4 wt%). These compositional differences indicate distinct fluxing and network-forming roles of the raw materials during thermal treatment, which is expected to strongly influence phase evolution and sintering behavior in the ceramic system. The chemical composition indicated by XRF analysis was further verified by Fourier-transform infrared (FTIR) spectroscopy (Fig. 4b). Infrared spectra were acquired using an FT-801 Fourier transform infrared spectrometer (Simex, Russia) configured with a single-reflection attenuated total reflectance (ATR) accessory featuring a ZnSe crystal. The acquisition parameters included a spectral resolution of 4 cm^−1^, and each final spectrum was produced by averaging 50 individual scans. The resulting FTIR spectra of the raw materials (Fig. 4b) reflect the oxide composition from XRF, displaying characteristic vibrational bands of the major functional groups (*e.g.*, Si–O, Al–O, C–O) present in the samples. The phase composition, as determined by X-ray diffraction (XRD) (Fig. 4a), was analyzed using a DRON-8 diffractometer (Burevestnik, Russia) with Cu-Kα radiation (*λ* = 1.5406 Å). Data collection spanned a 2*θ* range of 2–65° with a step size of 0.05°. XRD phase analysis reveals pronounced differences in the mineralogical composition of the raw materials (Fig. 5). WGBP is dominated by an amorphous SiO_2_ phase, CFA is primarily composed of mullite (75.3 wt%) and quartz (21.4 wt%) with minor magnetite (3.3 wt%), whereas SS consists mainly of Ca-bearing phases such as Ca-olivine (38.7 wt%) and mayenite (40.8 wt%) together with minor silicate and oxide phases. In contrast, CL exhibits a complex clay-rich assemblage dominated by kaolinite (38.5 wt%) and montmorillonite (25.3 wt%), accompanied by chlorite (13.6 wt%), quartz (9.3 wt%), and gypsum (8.1 wt%), reflecting its sedimentary origin and high mineralogical heterogeneity.

### 2.2 Preparation process

**Fig. 6:**
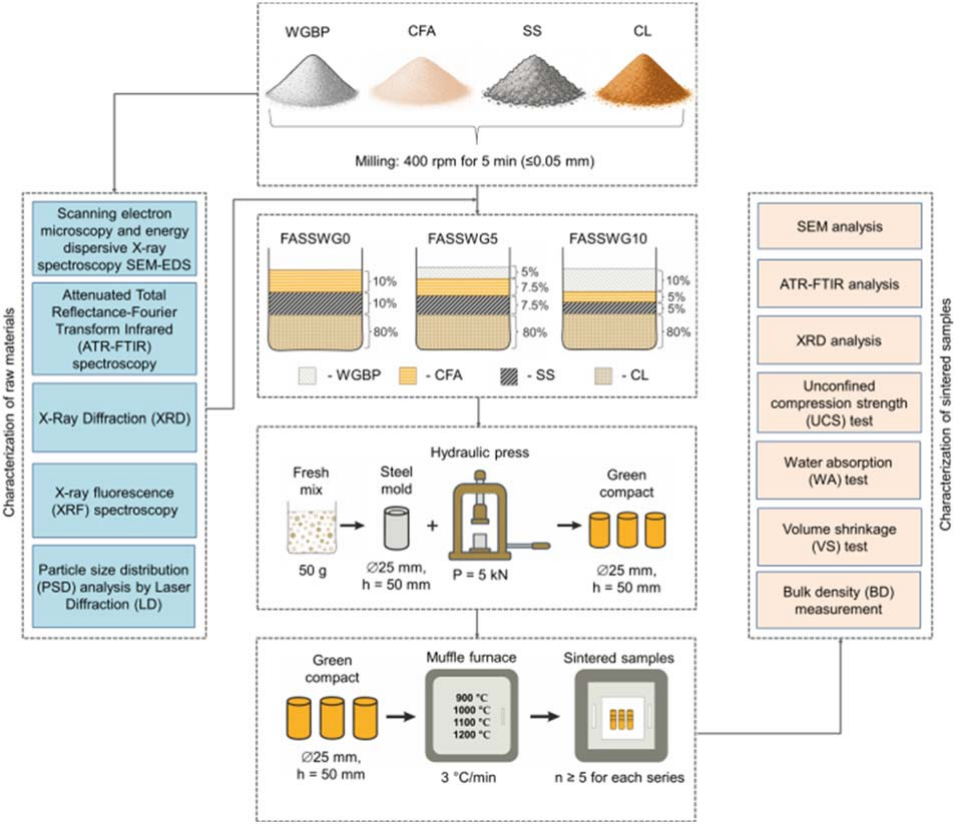
Schematic diagram of preparation process and characterization methods of ceramic samples.

To systematically evaluate the effect of waste glass powder addition, three mixture series were prepared with WGBP contents of 0, 5, and 10 wt%. In all compositions, the CL was maintained at a constant 80 wt% to ensure adequate plasticity and dimensional stability of the green bodies. The remaining 20 wt% consisted of SS and CFA in equal proportions, with their combined content decreasing as WGBP increased (Table 2). Each formulation (100 g dry mass) was mixed with 15 mL of deionized water for 10 minutes to ensure homogeneity. Green compacts were formed by uniaxially pressing 50 g aliquots of the moistened powder at 5 kN in a hydraulic press, with a dwell time of 60 seconds. The pressed cylindrical bodies were dried at 60 °C for 18 hours. Testing required two sample geometries: discs (25 mm diameter) for water absorption, volume shrinkage, contact angle and density measurements, and cylinders (25 mm diameter, 50 mm height) for compressive strength tests. A controlled sintering cycle was implemented in a muffle furnace LF-15/13-V11 (LOIP, Russia). Samples were heated from room temperature to the desired sintering point (900, 1000, 1100, or 1200 °C) at a rate of 3 °C min^−1^, held at that temperature for 6 hours, and finally allowed to cool down within the furnace to produce the final ceramic specimens. A schematic diagram of the overall preparation procedure is presented in Fig. 6.

**Table 2:** Raw material composition of ceramic formulations with varying solid waste incorporation

Formulation	CL (wt%)	SS (wt%)	CFA (wt%)	WGBP (wt%)	Firing conditions	
					Temperature (°C)	Time (h)
FASSWG0-900	80	10	10	0	900	6
FASSWG0-1000	80	10	10	0	1000	6
FASSWG0-1100	80	10	10	0	1100	6
FASSWG0-1200	80	10	10	0	1200	6
FASSWG5-900	80	7.5	7.5	5	900	6
FASSWG5-1000	80	7.5	7.5	5	1000	6
FASSWG5-1100	80	7.5	7.5	5	1100	6
FASSWG5-1200	80	7.5	7.5	5	1200	6
FASSWG10-900	80	5	5	10	900	6
FASSWG10-1000	80	5	5	10	1000	6
FASSWG10-1100	80	5	5	10	1100	6
FASSWG10-1200	80	5	5	10	1200	6

### 2.3 Test methods

Fourier Transform Infrared (FTIR) spectra were acquired using a Simeks FT-805 Fourier transform infrared spectrometer equipped (NPF SIMEX, Russia) with a zinc selenide attenuated total reflection (ATR) accessory. Spectra were recorded in the range of 500–4000 cm^−1^ at a resolution of 4 cm^−1^ by accumulating at least 50 scans. Prior to each measurement, a background spectrum of the clean ATR crystal was collected. For analysis, the powdered sample was pressed firmly onto the crystal surface. After each measurement, the crystal was thoroughly cleaned with ethanol-soaked cotton wool. The phase composition of the sintered materials was investigated by X-ray diffraction (XRD) using a Dron-8 diffractometer (Burevestnik, Russia) with Cu-Kα radiation (*λ* = 1.54 Å). Diffraction patterns were recorded in the 2*θ* range from 2° to 65° with a step width of 0.05° and a counting time of 3 seconds per point. The microstructure of the fracture surfaces of the fired ceramic bodies was examined using a Hitachi S3400n scanning electron microscope (SEM) operated in backscattered electron (BSE) mode at an accelerating voltage of 20 kV. Compressive strength tests were conducted on an IP-100 universal testing machine (ZIPO, Russia) with a 100 kN load cell at a constant loading rate of 0.15 MPa s^−1^. Water absorption was evaluated by immersing the specimens in deionized water at ambient conditions (23 ± 2 °C, 65 ± 5% RH) for 24 h. Finally, the bulk density was determined *via* the Archimedes (water immersion) method. Mass measurements for this procedure were performed with a BEL DA-1203C analytical balance (BEL Engineering, Italy) with a maximum capacity of 1200 g and a readability of 0.001 g. The compressive strength, bulk density, and water absorption of the fired samples were determined using eqn (1)–(3), respectively:1
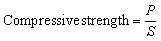
2
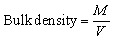
3
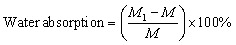
here, *P* (MN) is the maximum load recorded at the point of specimen fracture; *S* (m^2^) denotes the original cross-sectional area of the sample; *M* (g) is the final dry mass of the specimen after firing. Finally, *M*_1_ (g) is the mass measured after the fired specimen has been fully saturated with water *via* immersion.

## 3. Results and discussion

### 3.1 Physical indicators and mechanical properties

**Fig. 7:**
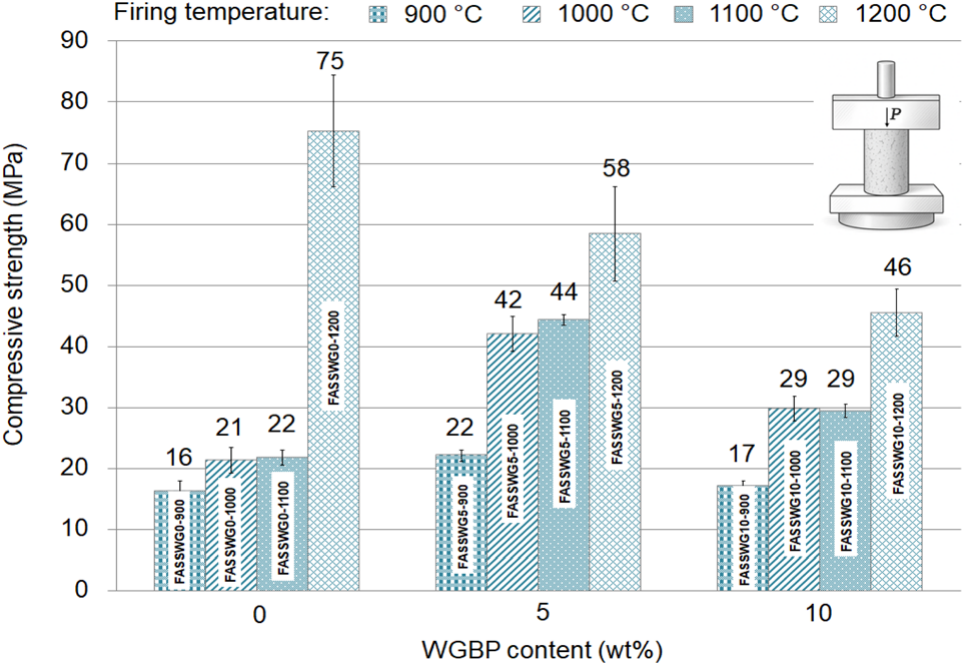
Evolution of compressive strength of the prepared ceramics as a function of WGBP addition and firing temperature.

Fig. 7 summarizes the compressive strength development of the tested ceramic mixtures as a function of firing temperature and WGBP dosage. Overall, strength increases with temperature for all formulations, confirming that densification and bond formation are strongly controlled by thermally activated sintering/vitrification processes. The UCS results agree with prior research on ceramics incorporating recycled waste glass materials.^36,37,44,45^ However, the effect of WGBP content is non-monotonic and depends on the sintering temperature, reflecting a transition from limited liquid-phase formation at lower temperatures to possible over-fluxing and microstructural heterogeneity at the highest temperature. At 900–1100 °C, the fired bodies exhibit moderate strengths (∼16–44 MPa), with the 5 wt% WGBP formulation consistently outperforming the WGBP-free and 10 wt% WGBP mixes. Specifically, at 1000–1100 °C the 5 wt% WGBP sample reaches ∼42–44 MPa, compared with ∼21–22 MPa for 0 wt% and ∼29 MPa for 10 wt% WGBP. This suggests that a limited amount of waste glass bottles-derived powder acts primarily as an efficient flux, promoting earlier onset of liquid-phase sintering, enhanced particle rearrangement, and accelerated pore closure.^43,44^ In this temperature range, the clay matrix supplies aluminosilicate frameworks, while SS and CFA likely contribute additional fluxing oxides and reactive phases,^47–50^ collectively supporting viscous flow and the formation of a more cohesive ceramic bond network.

The inferior performance at 10 wt% WGBP at the same temperatures implies that excessive glass content may increase the fraction of low-viscosity melt prematurely, which can trap gases,^43^ inhibit uniform densification, or generate a larger volume of glassy phase that is mechanically less favorable than a balanced crystalline-glassy microstructure.^51,52^ At 1200 °C the ranking reverses. The WGBP-free composition shows the maximum strength (∼75 MPa), whereas 5 wt% and 10 wt% WGBP reach ∼58 MPa and ∼46 MPa, respectively (Fig. 7). The sharp strength jump of the 0 wt% WGBP sample from ∼22 MPa (1100 °C) to ∼75 MPa (1200 °C) indicates that its densification threshold is reached primarily near 1200 °C. In contrast, WGBP-containing mixes likely enter a high liquid-phase fraction regime earlier; at 1200 °C this may result in over-fluxing, increased closed (entrapped) porosity, exaggerated grain growth, or thermal-elastic incompatibilities between crystalline inclusions and an expanded glassy matrix.^45,53^ Furthermore, increasing the waste glass bottles-derived powder content at higher firing temperatures (1200 °C) can lead to deformation and damage of the fired products due to blister formation on their surface, as also reported by Darweesh.^46^ Such features can reduce load-bearing cross-section and promote microcrack initiation, which would explain the systematically lower strengths at elevated WGBP dosages. Notably, the increase from 1100 to 1200 °C results in further strength enhancement for all formulations, consistent with continued densification.

**Fig. 8:**
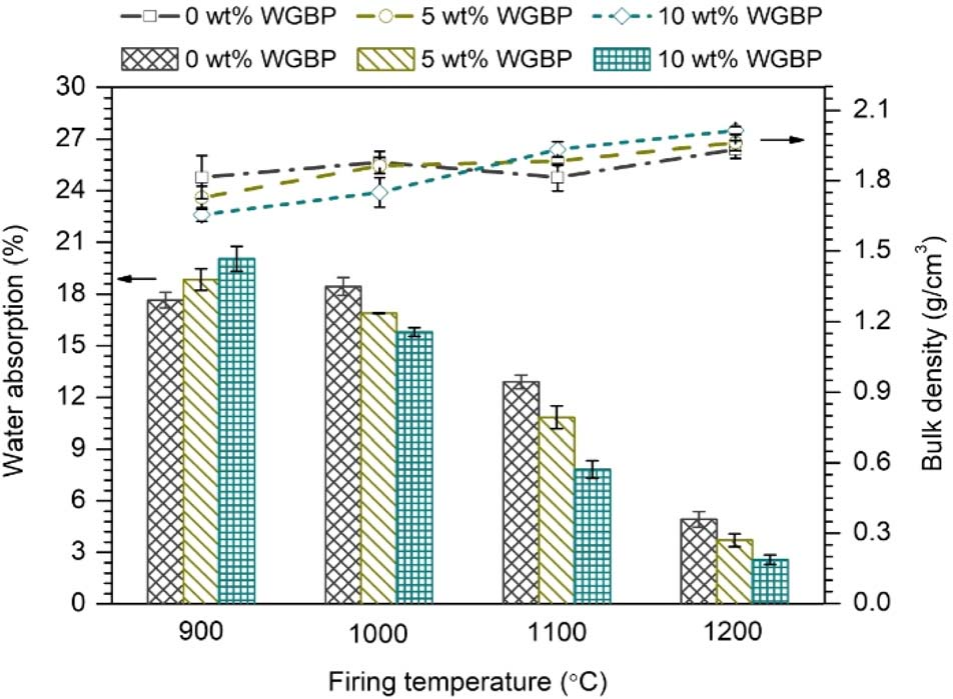
Evolution of water absorption and bulk density of the prepared ceramics as a function of WGBP addition and sintering temperature.

As shown by WA test results (Fig. 8), the water absorption values of all fired bodies do not exceed 20%, which is established as the limit for ceramic and porcelain tiles intended for use under extreme conditions according to ASTM C373 specifications. Furthermore, the samples fired at 1100 °C and 1200 °C meet the requirements of ASTM C62 specifications for building bricks, which specify a critical value of 17% for the amount of water absorbed. As shown in Fig. 8, water absorption decreases with increasing firing temperature for all compositions, indicating progressive densification and reduction of open porosity. At 900 °C, relatively high water absorption values (∼18–20%) are observed. At this temperature, the increased water absorption at 900 °C reflects the dominance of weakening and pore-forming processes over densification mechanisms. At 1000 °C, the influence of WGBP addition is still moderate; however, formulations containing WGBP (both 5 and 10 wt%) exhibit slightly lower water absorption than the ceramics without WGBP, indicating an earlier onset of liquid-phase sintering. A sharp decline in water absorption occurs at 1100–1200 °C, particularly for WGBP-containing ceramics. At 1200 °C, WA decreases to about 5% for the WGBP-free mixture and to approximately 3–4% for samples with 5–10 wt% WGBP (FASSWG5-1200 and FASSWG10-1200), confirming the role of waste glass in enhancing vitrification and sealing open pores at elevated temperatures. This low water absorption is attributed to the formation of a glassy surface at the higher sintering temperature of 1200 °C (see Section 3.2), which effectively prevents water penetration. This finding aligns with the observations reported by Huang *et al.*^45^

The evolution of bulk density (Fig. 8) mirrors the trends observed for water absorption and compressive strength. For all formulations, density increases with firing temperature, rising from ∼1.65–1.80 g cm^−3^ at 900 °C to ∼1.9–2.0 g cm^−3^ at 1200 °C. This reflects enhanced particle rearrangement, viscous flow, and solid–liquid phase interactions during firing. Adediran *et al.*^37^ also reported that the density of clay- and waste-based ceramics increased with sintering temperature when glass wool was used as a flux. At 1000–1100 °C, mixtures containing 5 wt% WGBP exhibit slightly higher density values. These results suggest that the presence of a limited amount of waste glass bottles-derived powder can facilitate densification in this temperature range, although the overall differences between formulations remain small. The progressive decrease in water absorption and increase in bulk density with firing temperature can be attributed to a well-defined sequence of reactions: dehydroxylation of clay minerals and formation of transient aluminosilicate intermediates (900–1000 °C), followed by mullite crystallization and softening of WGBP. Above 1000 °C, the Na_2_O–CaO–SiO_2_-rich melt promotes liquid-phase sintering, capillary-driven pore closure, and partial vitrification. Consistent with the phase-diagram structural approach to glass evolution,^54^ the melt acts as a mixture of nearest congruent compounds, and its redistribution during soaking and cooling governs the final balance between crystalline phases and residual glass, ultimately controlling densification.

Compared with the literature data (Table 3), this study demonstrates a more balanced combination of mechanical and physical properties. In particular, the combination of relatively high compressive strength (up to 58 MPa), reduced water absorption, and increased bulk density is maintained over an extended firing temperature range. Such robustness is advantageous for practical ceramic manufacturing and supports the applicability of the proposed compositions in resource-efficient construction materials.

**Table 3:** Summary of research results on physical and mechanical properties of ceramics incorporating recycled waste glass materials

Reference	55	56	57	This work
Waste glass content (wt%)	15–30	2.5–10	5–10	5–10
Firing temperature (°C)	1100	850–1050	900–1000	1000–1200
Compressive strength (MPa)	26–41	19–29	19–25	29–58
Water absorption (%)	2–3	11–19	15–19	3–17
Bulk density (g cm^−3^)	1.7–2.05	1.55–1.68	1.70–1.76	1.65–2.01

### 3.2 Phase composition and microstructure

**Fig. 9:**
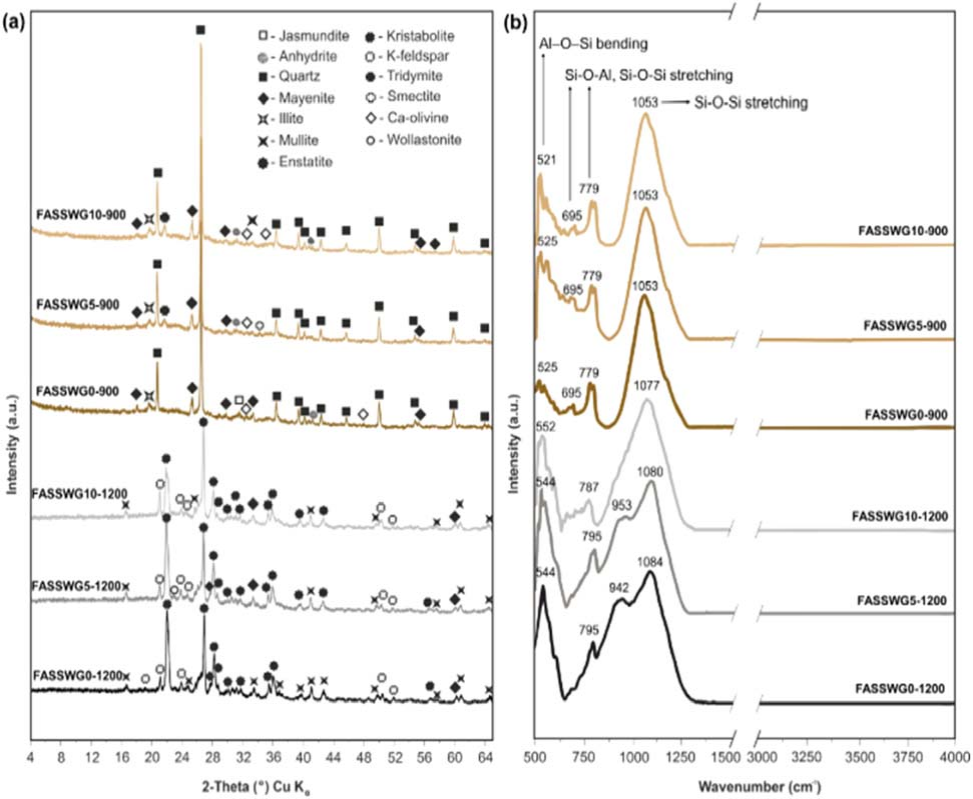
(a) XRD patterns and (b) FTIR spectrum for selected ceramic samples at different firing temperature (900 and 1200 °C) and WGBP dosage (0, 5 and 10 wt%).

**Fig. 10:**
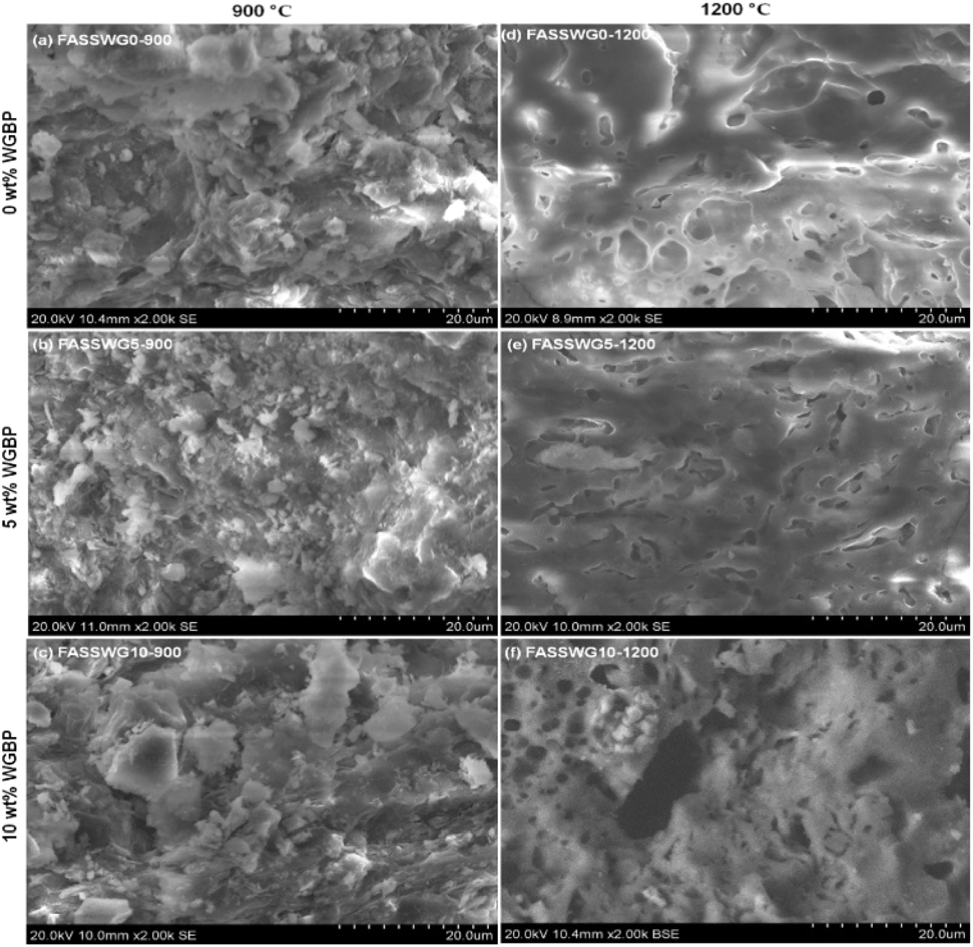
Fracture surface micrographs of selected ceramic samples at different firing temperature (900 and 1200 °C) and WGBP dosage (0, 5 and 10 wt%).

As shown in Fig. 9a, the XRD patterns at 900 °C (FASSWG0-900, FASSWG5-900, FASSWG10-900) are dominated by residual aluminosilicate and silicate phases, with clear reflections attributable to quartz and clay-derived phases (illite, smectite) alongside early high-temperature reaction products. The persistence of sharp quartz peaks indicates incomplete transformation of the initial framework at 900 °C, consistent with limited sintering and the relatively porous fracture textures observed by SEM (Fig. 10a–c). Minor crystalline calcium-bearing phases are also detectable, suggesting incipient reactions involving Ca-rich constituents (from SS/CFA and WGBP), but without extensive crystallization at this temperature. After firing at 1200 °C (FASSWG0-1200, FASSWG5-1200, FASSWG10-1200), the diffraction patterns evolve markedly, evidencing advanced high-temperature phase development. Peaks assigned to mullite become more pronounced, indicating intensified reactions within the aluminosilicate matrix.^58,59^ In parallel, the appearance and/or strengthening of reflections corresponding to high-temperature silicates (cristobalite/tridymite, feldspar-related phases, and Ca-bearing silicates such as wollastonite) point to a more extensive restructuring of the system and stronger participation of Ca–Si–Al reactions during firing.^60,61^ Importantly, the relative prominence of crystalline peaks *versus* the implied amorphous contribution suggests that vitrification is significant at 1200 °C, and the degree of crystallinity likely reflects a balance between melt formation and crystallization. With increasing WGBP dosage, the patterns remain broadly similar in phase assemblage, but the microstructural evidence (Fig. 10d–f) suggests that changes in melt amount and its distribution may become more decisive than phase identity alone for the final performance.

The FTIR spectra provide complementary evidence of thermally driven structural reorganization (Fig. 9b). At 900 °C, the spectra retain strong bands associated with aluminosilicate frameworks, including Si–O–(Si,Al) stretching in the 1000–1100 cm^−1^ region and Al–O–Si bending features in the 520–560 cm^−1^ range.^62–64^ The presence of bands near 695 and 779 cm^−1^ is consistent with quartz-related vibrations,^59,65,66^ aligning with the persistence of quartz reflections in XRD. Notably, the main Si–O stretching envelope shows little to no shift at 900 °C, indicating that the silicate network remains largely unchanged and insufficiently reorganized into a dense vitrified matrix. At 1200 °C, the spectra show a clearer signature of network reconfiguration and vitrification. Structural disorder in the tetrahedral sheets is reflected through a broadening of the characteristic Si–O stretching vibration band around 1000–1100 cm^−1^.^60^ The Si–O stretching region becomes broader and shifts in position/intensity (with features near 1080–1084 cm^−1^ and the emergence/strengthening of bands around 942–953 cm^−1^), which is consistent with the formation of a more developed silicate network and/or increased contribution of Ca-modified silicate structures typical of liquid-phase sintering products.^58,60,67^

The fracture micrographs at 900 °C display a predominantly under-sintered morphology for all dosages, characterized by rough, angular fracture surfaces, partially bonded particles, and a visibly open pore network (Fig. 10a–c). The WGBP-free mixture (FASSWG0-900) shows heterogeneous particle packing and weak interparticle bonding, consistent with incomplete densification. The mixture with 5 wt% WGBP (FASSWG5-900), the surface appears slightly more consolidated and finer in texture, suggesting some enhancement of particle bonding; however, porosity remains evident. At 10 wt% WGBP (FASSWG10-900), larger plate-like fragments and a more heterogeneous fracture topology are observed, implying that at 900 °C the additional glass does not yet translate into a uniformly sintered matrix and may instead accentuate local heterogeneity in bonding and packing.

At 1200 °C, all samples transition toward a viscously sintered fracture morphology, with a smoother matrix and more rounded pores indicative of extensive liquid-phase participation (Fig. 10d–f). The WGBP-free mixture (FASSWG0-1200) exhibits a strongly densified glass-ceramic-like texture but also contains rounded, bubble-like voids, suggesting gas entrapment during vitrification. The mixture with 5 wt% WGBP (FASSWG5-1200) appears comparatively more uniform, with a continuous matrix and fewer large defects, consistent with controlled liquid-phase sintering and improved pore sealing.^38,57,59^ In contrast, the mixture with 10 wt% WGBP (FASSWG10-1200) shows a microstructure with more abundant and/or larger rounded pores and local heterogeneity, which is compatible with over-fluxing behavior.

### 3.3 Sustainability assessment

To provide a general evaluation of the environmental performance of the developed ceramic compositions, a simplified quantitative sustainability assessment was carried out. The analysis combined mechanical test results with average estimates of firing energy and associated CO_2_ emissions, representing the dominant environmental burden in ceramic production. The specific energy demand of tunnel kilns was assumed to increase linearly with firing temperature, starting from 2.8 MJ kg^−1^ at 900 °C and rising by approximately 0.15 MJ kg^−1^ for each additional 100 °C, based on reported industrial data.^67–71^ Corresponding CO_2_ emissions were calculated using the emission factor of 0.056 kg CO_2_/MJ for natural gas combustion according to IPCC Guidelines for National Greenhouse Gas Inventories.^72^ The energy-normalized strength (*N* = *f*_c_/*E*) was then used as an eco-efficiency indicator that links the achieved compressive strength (*f*_c_) to the required firing energy (*E*). This approach allows direct comparison between mixtures with different waste contents and firing regimes, quantifying how improvements in strength and reductions in temperature jointly influence the overall energy efficiency and carbon footprint of the ceramic process. It should be emphasized that the present calculation is intended as a comparative process-level assessment and does not constitute a full life-cycle analysis.

The energy-normalized strength (Table 4) indicates that ceramic mixture FASSWG5 (5 wt% WGBP) retains the highest eco-efficiency at 1000–1100 °C (14 MPa kg MJ^−1^), which is about twice the value of the reference mixture FASSWG0 at the same temperature (7 MPa kg MJ^−1^). The reference formulation achieves its maximum efficiency (23 MPa kg MJ^−1^) only at 1200 °C, where both the energy demand and CO_2_ emissions are highest. From a sustainability standpoint, the reference ceramic mixture's very high strength at 1200 °C is likely over-specified for typical units, offering limited benefit *versus* its extra energy and carbon emissions. Ceramic mixture FASSWG10 (10 wt% WGBP) provides intermediate efficiency, highlighting a trade-off between higher waste utilization and slight loss in mechanical performance without receiving the same fluxing benefit from WGBP as the FASSWG5 mixture does.

**Table 4:** Sustainability assessment of the tested ceramic mixture

Formulation	*f*_c_ (MPa)	*T* (°C)	*E* (MJ kg^−1^)	CO_2_ emissions (kg kg^−1^)	*N* (MPa kg MJ^−1^)
FASSWG0-900	16	900	2.80	0.157	5.71
FASSWG0-1000	21	1000	2.95	0.165	7.12
FASSWG0-1100	22	1100	3.10	0.174	7.10
FASSWG0-1200	75	1200	3.25	0.182	23.08
FASSWG5-900	22	900	2.80	0.157	7.86
FASSWG5-1000	42	1000	2.95	0.165	14.24
FASSWG5-1100	44	1100	3.10	0.174	14.19
FASSWG5-1200	58	1200	3.25	0.182	17.85
FASSWG10-900	17	900	2.80	0.157	6.07
FASSWG10-1000	29	1000	2.95	0.165	9.83
FASSWG10-1100	29	1100	3.10	0.174	9.35
FASSWG10-1200	46	1200	3.25	0.182	14.15

In general, the simplified sustainability assessment (Table 4) indicates that the ceramic mixture FASSWG5-1000 with 5 wt% WGBP fired at 1000 °C emerges as the most sustainable formulation for load-bearing construction applications. It incorporates a significant fraction of industrial by-products, including waste glass bottles-derived powder, achieves compressive strengths of 42 MPa that exceed those of the reference formulation at the same firing temperatures and comfortably satisfy building-code requirements, and enables a reduction in firing temperature by at least 200 °C relative to the 1200 °C reference route, implying a notable decrease in fuel consumption and kiln-related CO_2_ emissions. Ceramic mixture FASSWG10 with 10 wt% WGBP offers the highest degree of waste utilization and reaches 29 MPa at 1000–1100 °C, which could be attractive for non-load-bearing units and blocks in low-rise construction, where mechanical requirements are less stringent and waste diversion is prioritized. The reference ceramic mixture without WGBP requires higher temperatures to reach competitive strength and thus represents the least favorable option in terms of energy and carbon dioxide emissions.

## 4. Conclusions

This study demonstrates the feasibility of producing sustainable, high-performance construction ceramics through the integrated utilization of kaolinitic clay, steel slag, coal fly ash, and recycled waste glass bottles-derived powder. Based on the experimental results, the following conclusions can be drawn:

(1) Increasing the firing temperature from 900 to 1200 °C leads to progressive densification, reflected by increased compressive strength and bulk density, as well as reduced water absorption for all compositions. Ceramics fired at 1100–1200 °C satisfy relevant ASTM limits for building bricks and tiles, with water absorption reduced to 3–5% and bulk density reaching up to 2.01 g cm^−3^.

(2) At intermediate firing temperatures (1000–1100 °C), the addition of 5 wt% WGBP effectively promotes liquid-phase sintering, resulting in improved microstructural uniformity, enhanced pore closure, and compressive strengths up to 44 MPa. At 1200 °C, higher WGBP contents induce excessive vitrification, pore entrapment, and microstructural heterogeneity, which negatively affect mechanical performance.

(3) XRD and FTIR analyses reveal a transition from residual aluminosilicate and quartz-rich assemblages at 900 °C to mullite-bearing, partially vitrified glass-ceramic systems at 1200 °C. SEM fracture surface observations confirm the evolution from under-sintered, porous morphologies to viscously sintered structures, with optimal microstructural homogeneity observed for the 5 wt% WGBP composition.

(4) Energy-normalized strength analysis indicates that the ceramic composition containing 5 wt% WGBP fired at 1000–1100 °C offers the most favorable balance between mechanical performance and firing energy demand, enabling a reduction in firing temperature by up to 200 °C and a corresponding decrease in CO_2_ emissions (9–12%) relative to the 1200 °C reference route.

Overall, the proposed ceramic system provides a robust and resource-efficient pathway for valorizing multiple industrial waste streams into construction materials, supporting circular economy principles while maintaining competitive technical performance.

## Author contributions

Madeniyet Yelubay: conceptualization, methodology, investigation, data curation, formal analysis, validation, visualization, writing – original draft, writing – review & editing. Dias Tolegenov: investigation, data curation, formal analysis, visualization, writing – review & editing. Sabit Maussumbayev: conceptualization, methodology, formal analysis, investigation, resources, software, validation, visualization, supervision, project administration, writing – original draft, writing – review & editing. Nurdana Kanasheva: methodology, resources, software, validation, writing – review & editing. Gulzat Aitkaliyeva: investigation, data curation, resources, validation, writing – review & editing. Vladimir Mokichev: methodology, formal analysis, software, validation, writing – review & editing. Stepan Denisov: methodology, resources, software, validation, writing – review & editing. Sergey Tsvetkov: formal analysis, software, validation, visualization, writing – review & editing. Anton Kasprzhitskii: conceptualization, methodology, supervision, validation, writing – review & editing. Georgy Lazorenko: conceptualization, supervision, project administration, resources, validation, writing – review & editing.

## Conflicts of interest

There are no conflicts to declare.

## Data Availability

The data supporting the findings of this study are included within the article and its figures and tables. No additional datasets were generated or analysed during the current study.
